# Supplementary material for: The Cannabis Plant as a Complex System: Interrelationships between Cannabinoid Compositions, Morphological, Physiological and Phenological Traits
Source: Plants (Basel). 2023 Jan 21;12(3):493. doi: 10.3390/plants12030493 (PMC9919051; doi:10.3390/plants12030493)
Supplement: Supplementary file 1 [file plants-12-00493-s001.zip › Supplementary/Figure S1- Spatial adjustments.pdf]

CBC content

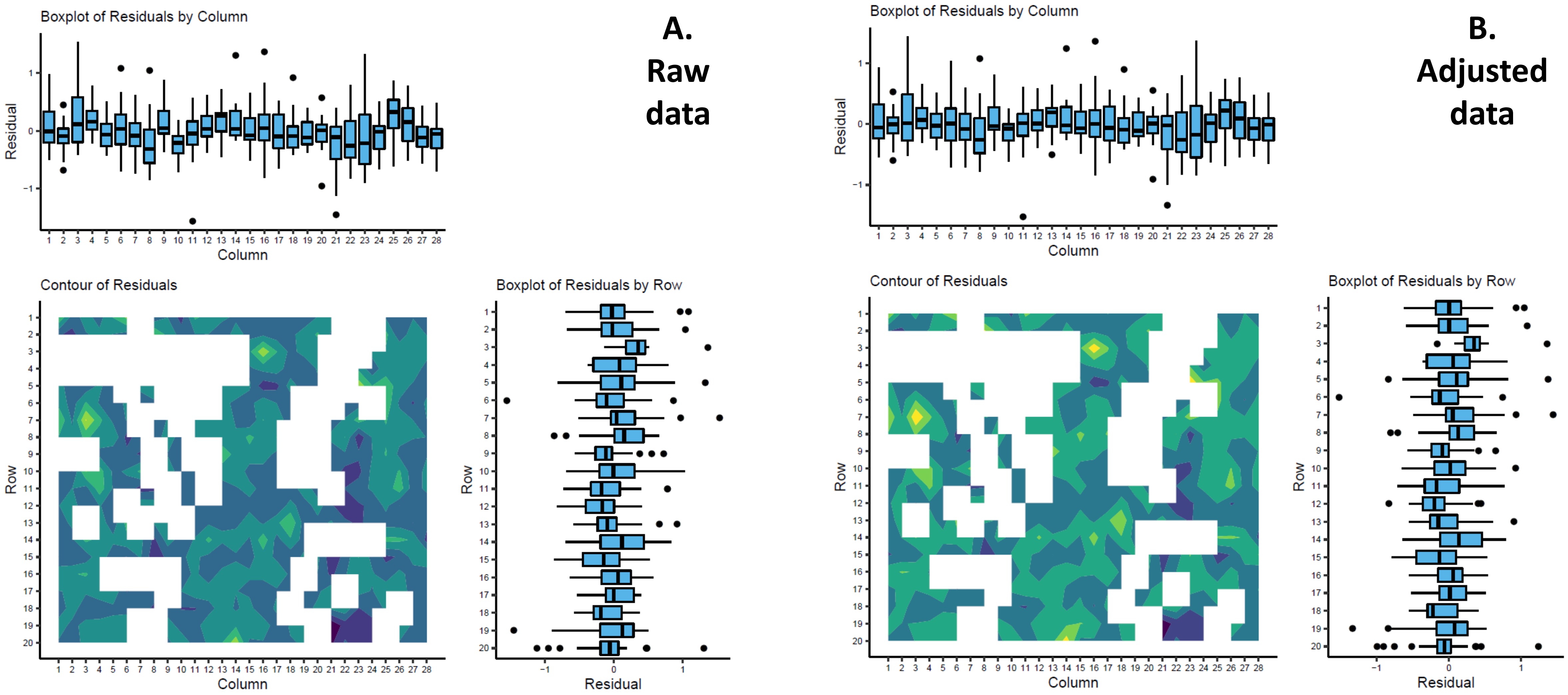

Figure S1- 1: A graphical representation of environmental variation within the controlled environment (CE) facility and the applied spatial adjustments. A: Raw data (prior to adjustments) which includes a heatmap for environmental variation across all positions within the CE facility as well as boxplots of the residuals to reflect the effect of environmental variations between rows and between columns for CBC content. B: Adjusted data (after spatial adjustments) which includes a heatmap for environmental variation across all positions within the CE facility as well as boxplots of the residuals to reflect the effect of spatial adjustments on CBC content. Blank areas in the heatmaps represent missing data

CBD content

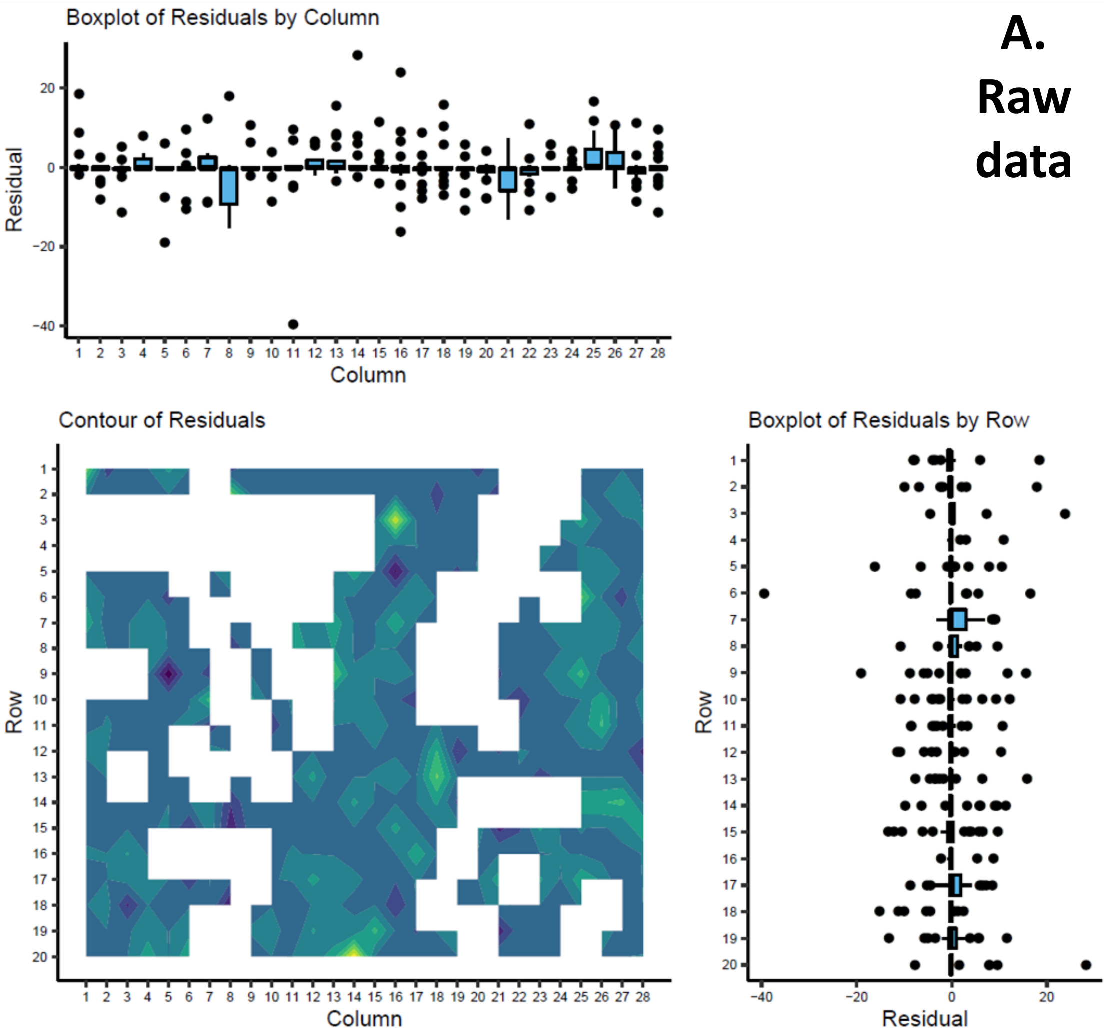

A. Raw data

Figure S1- 2: A graphical representation of environmental variation within the controlled environment (CE) facility. Raw data is depicted via a heatmap for environmental variation across all positions within the CE facility as well as boxplots of the residuals to reflect the row and column position effect for CBD. Blank areas in the heatmaps represent missing data. Spatial adjustments for CBD were not required and therefore were not conducted

CBDV content

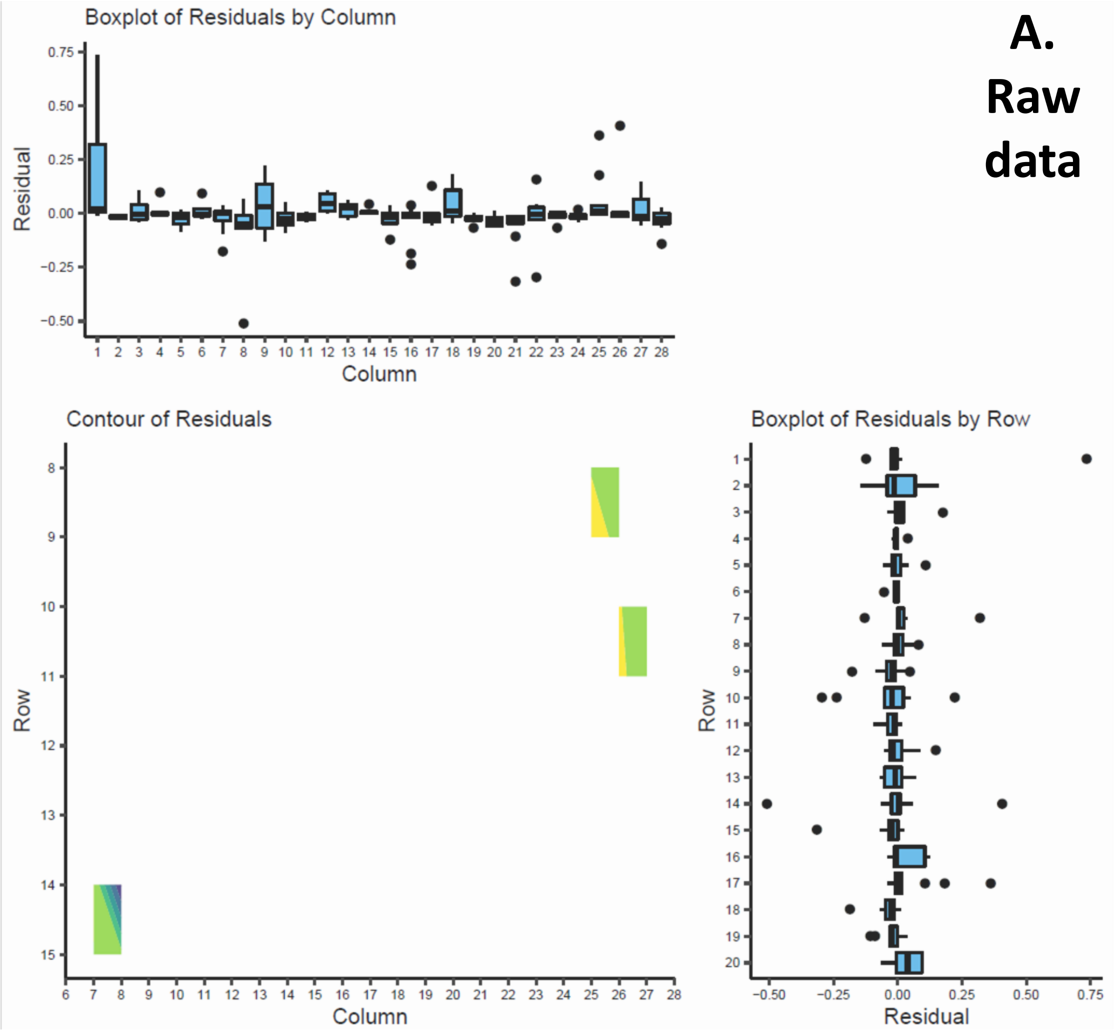

Figure S1- 3: A graphical representation of environmental variation within the controlled environment (CE) facility. Raw data is depicted via a heatmap for environmental variation across all positions within the CE facility as well as boxplots of the residuals to reflect the row and column position effect for CBDV. Blank areas in the heatmaps represent missing data. Spatial adjustments for CBDV were not required and therefore were not conducted

CBG content

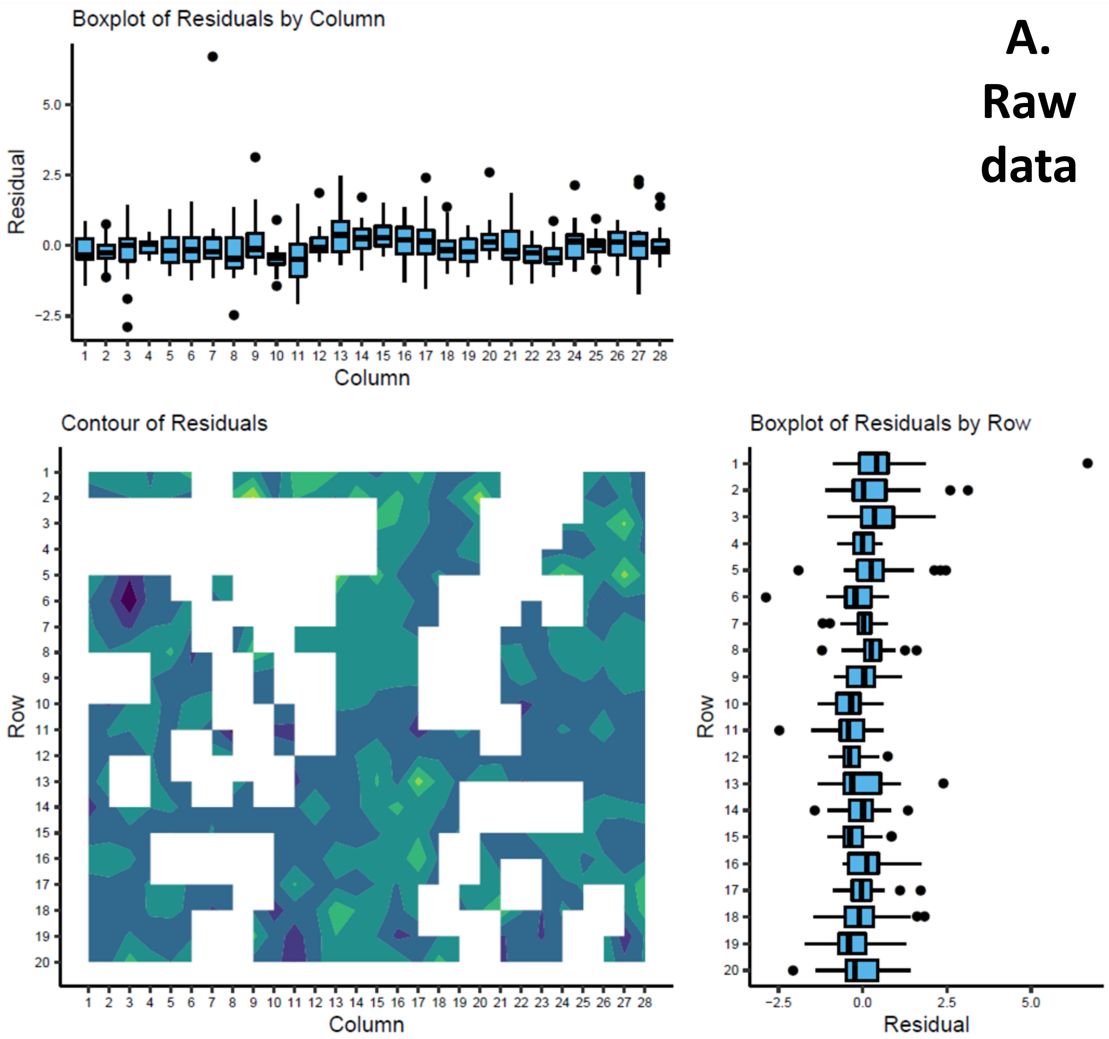

A. Raw data

Figure S1- 4: A graphical representation of environmental variation within the controlled environment (CE) facility. Raw data is depicted via a heatmap for environmental variation across all positions within the CE facility as well as boxplots of the residuals to reflect the row and column position effect for CBG. Blank areas in the heatmaps represent missing data. Spatial adjustments for CBG were not required and therefore were not conducted

OFFICIAL

CBN content

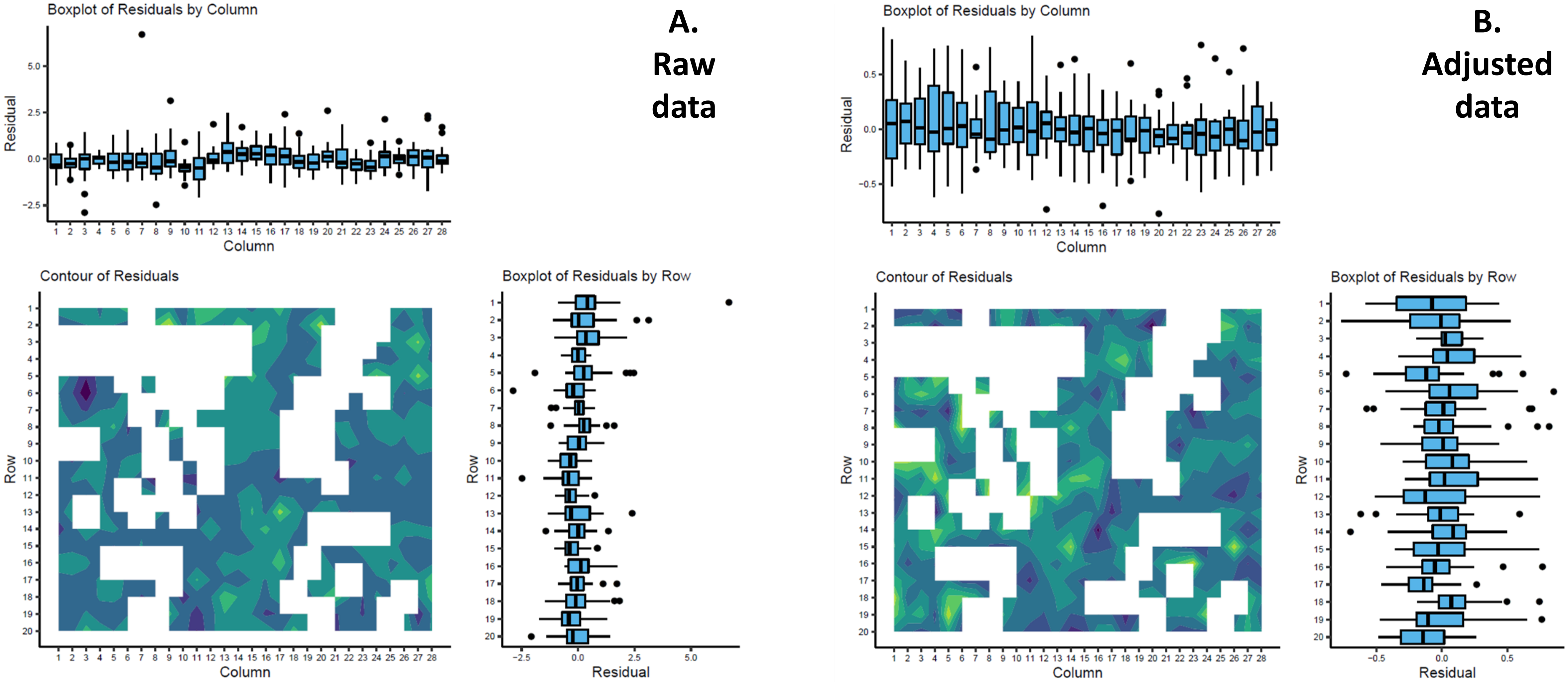

Figure S1- 5: A graphical representation of environmental variation within the controlled environment (CE) facility and the applied spatial adjustments. A: Raw data (prior to adjustments) which includes a heatmap for environmental variation across all positions within the CE facility as well as boxplots of the residuals to reflect the effect of environmental variations between rows and between columns for CBN content. B: Adjusted data (after spatial adjustments) which includes a heatmap for environmental variation across all positions within the CE facility as well as boxplots of the residuals to reflect the effect of spatial adjustments on CBN content. Blank areas in the heatmaps represent missing data

OFFICIAL

OFFICIAL

Total Cannabinoids Concentration

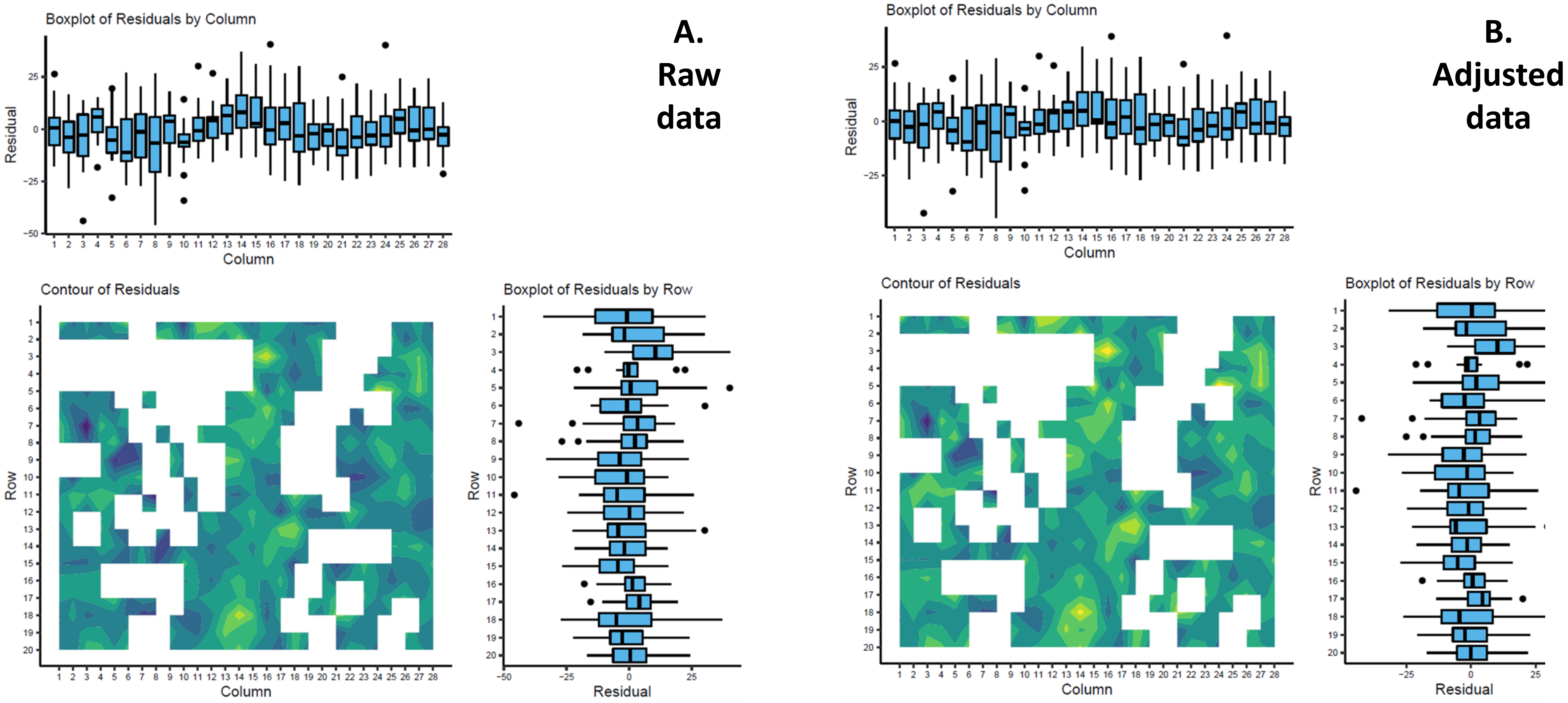

Figure S1- 6: A graphical representation of environmental variation within the controlled environment (CE) facility and the applied spatial adjustments. A: Raw data (prior to adjustments) which includes a heatmap for environmental variation across all positions within the CE facility as well as boxplots of the residuals to reflect the effect of environmental variations between rows and between columns for Total Cannabinoids Concentration. B: Adjusted data (after spatial adjustments) which includes a heatmap for environmental variation across all positions within the CE facility as well as boxplots of the residuals to reflect the effect of spatial adjustments on Total Cannabinoids Concentration. Blank areas in the heatmaps represent missing data

OFFICIAL

OFFICIAL

THC content

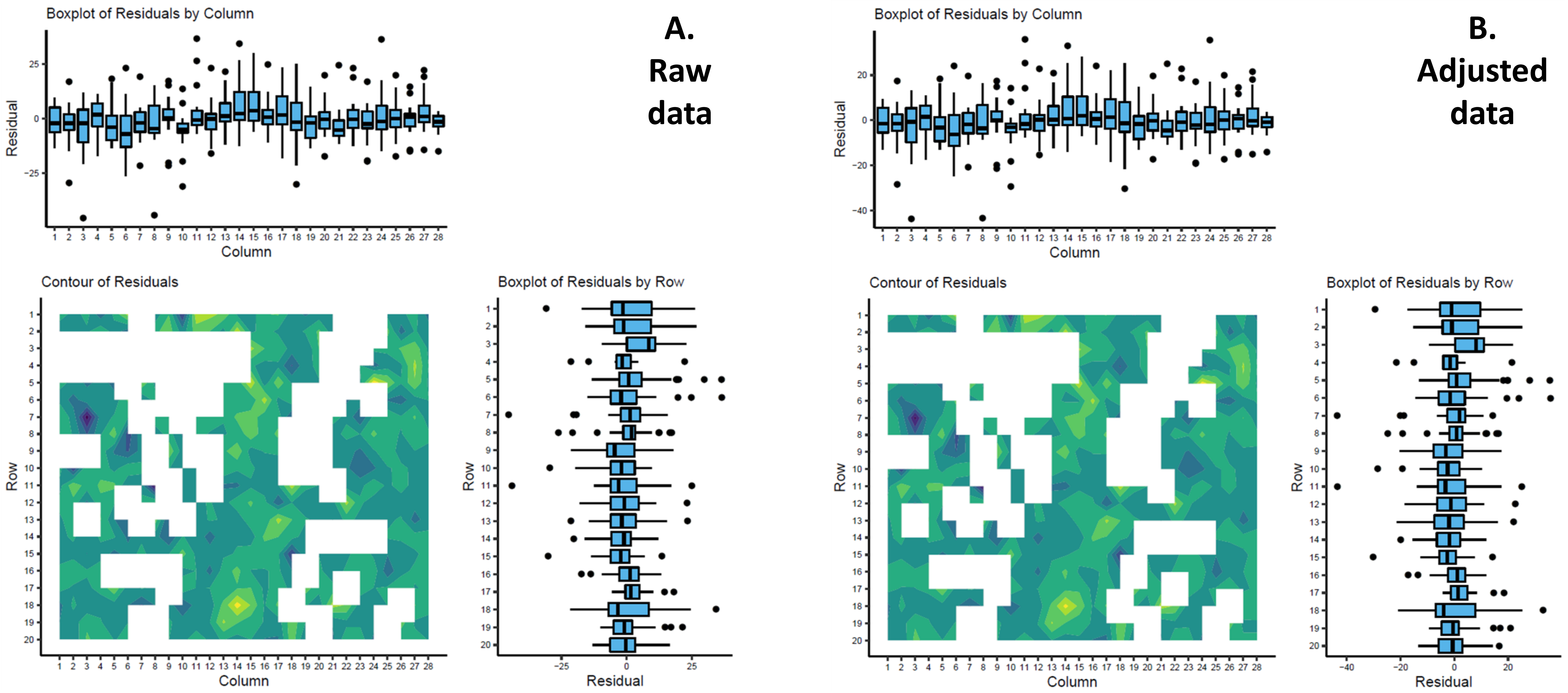

Figure S1- 7: A graphical representation of environmental variation within the controlled environment (CE) facility and the applied spatial adjustments. A: Raw data (prior to adjustments) which includes a heatmap for environmental variation across all positions within the CE facility as well as boxplots of the residuals to reflect the effect of environmental variations between rows and between columns for THC content. B: Adjusted data (after spatial adjustments) which includes a heatmap for environmental variation across all positions within the CE facility as well as boxplots of the residuals to reflect the effect of spatial adjustments on THC content. Blank areas in the heatmaps represent missing data

OFFICIAL

OFFICIAL

THCV content

A. Raw data

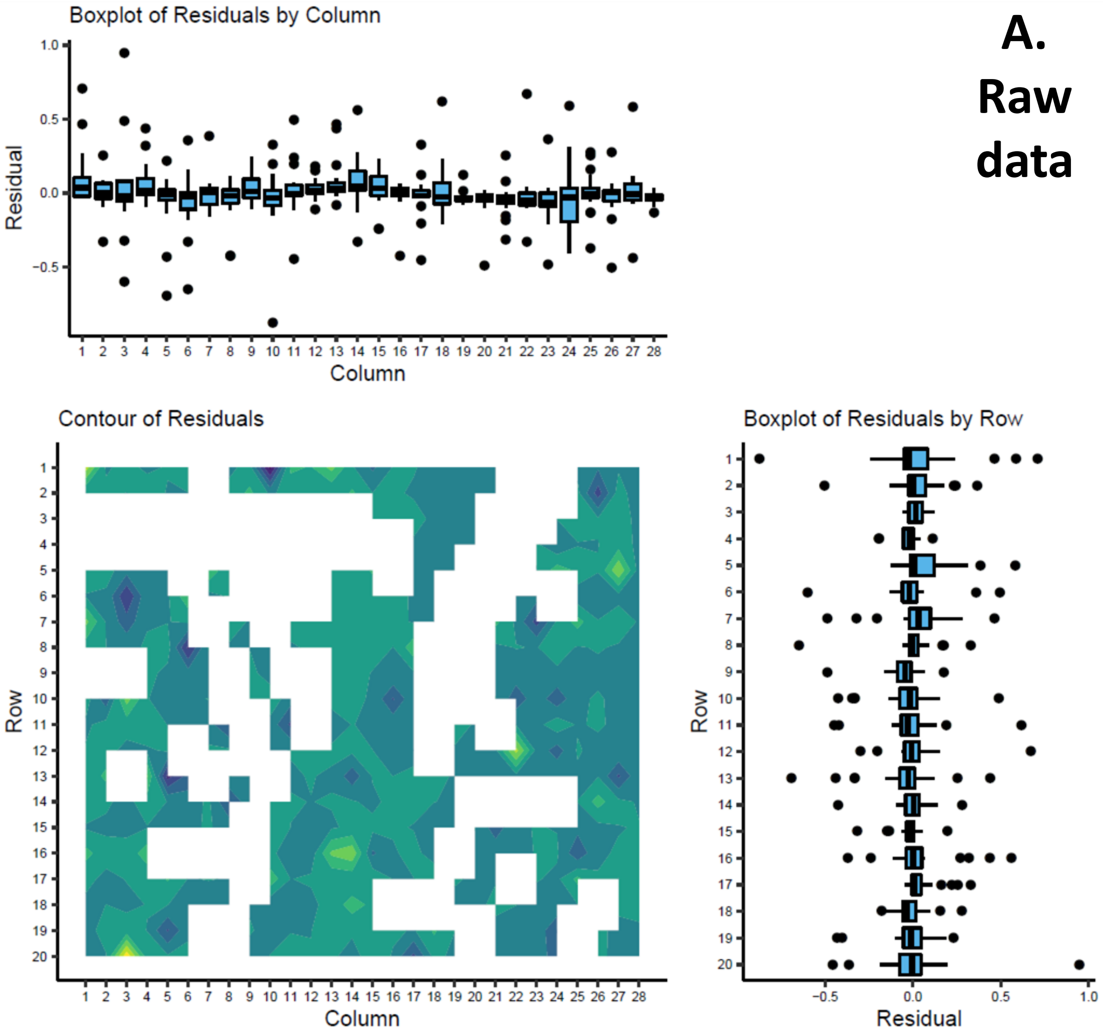

Figure S1- 8: A graphical representation of environmental variation within the controlled environment (CE) facility. Raw data is depicted via a heatmap for environmental variation across all positions within the CE facility as well as boxplots of the residuals to reflect the row and column position effect for THCV. Blank areas in the heatmaps represent missing data. Spatial adjustments for THCV were not required and therefore were not conducted

OFFICIAL
